# Effect of *Litsea cubeba* and Cinnamon Essential Oil Nanoemulsion Coatings on the Preservation of Plant-Based Meat Analogs

**DOI:** 10.3390/foods13213365

**Published:** 2024-10-23

**Authors:** Yiqun Zhu, Mengqing Gu, Yuhan Su, Zhe Li, Tiemin Xie, Yifan Zhang, Guohua Qiao, Fei Lu, Chunyang Han

**Affiliations:** 1 College of Grain Science and Technology, Shenyang Normal University, Shenyang 110034, China; 2 Shenyang Key Laboratory of Grain and Oil Deep Processing, Shenyang 110034, China; 3 School of Food and Biological Engineering, Hezhou University, Hezhou 542899, China

**Keywords:** cinnamon essential oil, *Litsea cubeba* essential oil, nanoemulsion stability, plant-based meat analog, preservation, quality attribute

## Abstract

Plant-based meat analogs (PBMAs) are promising sustainable food sources. However, their high moisture and protein contents make them prone to microbial deterioration, limiting their shelf life and sensory appeal. This study explored enhancing PBMAs’ shelf life using nanoemulsions of *Litsea cubeba* and cinnamon essential oils, emulsified with chitosan and Tween 80. The composite nanoemulsion, produced through high-pressure homogenization, exhibited a droplet size of 4.99 ± 0.03 nm, a polydispersity index (PDI) of 0.221 ± 0.008, and a zeta potential of 95.13 ± 2.67 mV, indicating remarkable stability (*p* < 0.05). Applied to PBMAs stored at 4 °C, it significantly improved color and pH balance and reduced thiobarbituric acid reactive substances and cooking loss. Most notably, it inhibited the growth of *Escherichia coli* and *Staphylococcus aureus*, curbing spoilage and protein oxidation, thereby extending the products’ shelf life and preserving sensory quality. As shown above, the encapsulation of LCEO/CEO in nanoemulsions effectively inhibits spoilage and deterioration in PBMAs, improving flavor and quality more than direct addition. Future studies should explore using various essential oils and emulsifiers, as well as alternative encapsulation techniques like microcapsules and nanoparticles, to further prevent PBMA deterioration.

## 1. Introduction

Plant-based meat analogs (PBMAs) are becoming a crucial advancement in the food sector, particularly with an expected world population of 9–10 billion by 2050, which will significantly increase protein requirements [1]. These innovative products encompass a diverse range of alternatives, including but not limited to plant-based iterations of burgers, sausages, and poultry substitutes, which are designed to emulate the organoleptic properties and nutritional profiles of their animal-based counterparts while offering enhanced sustainability [2,3,4]. PBMAs, which typically consist of 50–80% water, 15–30% plant proteins, and 5–15% lipids, are created using sophisticated manufacturing methods, such as extrusion, electrospinning, 3D printing, and shearing [5,6]. These approaches imitate the texture and flavor of animal proteins. High-moisture extrusion is particularly efficient in the production of meat-like fibrous structures, as it enables the manipulation of the moisture, temperature, and pressure of the feed [7]. However, elevated moisture and protein levels in PBMAs may facilitate the growth of microorganisms, causing spoilage owing to oxidation and putrefaction. This can cause the development of unpleasant aromas and smells and texture decline [8]. This presents a significant challenge, emphasizing the need for improved and long-lasting preservation techniques. Various strategies have been used in PBMA preservation, including low-temperature treatments, coating procedures, and the application of natural preservatives [8,9,10,11]. These approaches are promising in prolonging the shelf life and preserving the overall quality of PBMAs; however, implementing and assessing PBMAs in real-world situations remain challenging. While many preservation methods used for animal meats, such as modified atmosphere packaging, irradiation, and high-pressure processing, can potentially be applied to PBMAs, their efficacy and impact on the unique composition and structure of plant-based products require further investigation. This prompts an investigation into reliable and efficient techniques to guarantee the overall excellence of PBMAs.

Researchers are investigating essential oils as natural preservatives to bridge the gap between the impressive longevity of novel PBMAs and their limited shelf life, typically ranging from a few days to several weeks. *Litsea cubeba*, an indigenous plant in China and a member of the Camphoraceae family, is renowned for its essential oil (*Litsea cubeba* essential oil, LCEO), which has been recognized as Generally Recognized as Safe (GRAS) by the U.S. Food and Drug Administration (FDA) for use as a flavoring substance. It is also approved for use as a food additive in various countries, including China, Japan, and several European nations, though regulatory status may vary globally [12,13]. Analysis of LCEO reveals a complex mixture of compounds, primarily divided into three categories based on their isoprene unit content: monoterpenes (C_5_H_8_)_2_, sesquiterpenes (C_5_H_8_)_3_, and others. The monoterpenes compounds are the most abundant. In general, the oxygenated monoterpene, citral, is the most abundant component (50–90%) of the essential oil of the fruits of LC. It can be further classified into geranial and nerolidol due to the difference in the cis–trans isomerization of the carbon–carbon double bond in its structure. Most of the studies proved that geranial was higher than nerolidol. The content of the remaining components, such as monoterpene hydrocarbons like limonene, citronellal, geraniol, linalool, methyl heptenone, and other fractions, is influenced by various factors [14,15,16]. This oil exhibits effective antifungal and antioxidative characteristics, which particularly benefits safeguarding meat products by impeding the proliferation of harmful fungi such as *Aspergillus flavus*, *Penicillium expansum*, and *Fusarium graminearum* [17]. Although LCEO is effective, its use is hindered by its unpleasant odor, limited solubility in water, tendency to evaporate, and lack of stability, particularly under thermal stress and exposure to oxygen [18]. Similarly, cinnamon, belonging to the family Lauraceae and grown in several locations, produces an essential oil (cinnamon essential oil, CEO) rich in cinnamaldehyde. Cinnamaldehyde is well known for its antioxidant, anti-inflammatory, and strong antibacterial properties [19]. Gas chromatography–mass spectrometry analysis identified 13 compounds in CEO, accounting for 97.96% of the total area. Cinnamaldehyde was the dominant compound, with the highest peak area percentage of 92.40%. Trans-cinnamaldehyde was the second most significant compound, at 2.73%, followed by benzaldehyde at 1.52%. Other compounds, such as styrene, diacetone alcohol, benzylcarboxaldehyde, phenol, trans-cinnamic acid, and octadecadienoic acid, were present in trace amounts [20]. LCEO and CEO are limited in real-world implementation owing to their inherent physical characteristics. Nanoemulsion systems provide a means of encapsulating these oils, causing improved solubility, stability, and efficacy. The nanoemulsions, owing to their high specific surface area, improve the accessibility to active substances and are excellent carriers of antifungal components. This offers a viable and sustainable method for food preservation, which is crucial for ensuring food safety and quality in the future [21].

The stability of nanoemulsions is crucial for their commercial use, with emulsifiers being vital in preserving this stability [22]. Amphiphilic molecules, including tiny surfactants, proteins, polysaccharides, phospholipids, and other surface-active polymers, are crucial in stabilizing liquids that mix partially [22]. Chitosan and Tween 80 are efficient emulsifiers in nanoemulsion compositions, enhancing stability whether used alone or combined. For instance, chitosan has been successfully used in stabilizing curcumin nanoemulsions for enhanced bioavailability [23], while Tween 80 has shown effectiveness in creating stable nanoemulsions of essential oils for antimicrobial food packaging [24]. A combination of several emulsifiers usually causes a more stable emulsion than a single emulsifier [25]. This has significant advantages in food preservation, as it helps maintain the effectiveness and durability of the encapsulated antibacterial and antioxidative substances.

In this study, high-pressure homogenization was used to prepare nanoemulsions of *Litsea cubeba* and cinnamon essential oils, using chitosan and Tween 80 as co-emulsifiers. Furthermore, a nanoemulsion containing both essential oils was developed. This study was used to characterize nanoemulsions by analyzing their particle size, polydispersity index (PDI), zeta potential, and the ability of emulsifiers to stabilize them. In addition, this study was used to investigate the effects of the LCEO and CEO nanoemulsions and their combined nanoemulsion on the preservation of PBMAs. An analysis was conducted to identify and examine the pH, color, cooking loss rate, thiobarbituric acid reactive substances (TBARs), microbes, and protein secondary structure of the PBMAs throughout various storage periods. These results demonstrate a pioneering method for the preservation of PBMAs by synergistically incorporating *Litsea cubeba* and cinnamon essential oils into a stabilized nanoemulsion. This research represents the inaugural attempt at merging these essential oils and utilizing them for the preservation of plant-based meat analogs. The innovative formulation significantly enhances the durability of PBMAs, presenting optimal preservation conditions, which may also be applicable to the future development of cultured meat products.

## 2. Materials and Methods

### 2.1. Materials and Reagents

LCEO and CEO were acquired from Hangzhou Tianyi Fragrance Fine Co., Ltd. (Hangzhou, China). The manufacturer stated that the essential oil (EO) was obtained through hydrodistillation. Chitosan (deacetylation: 92%, molecular weight: 4.0 × 10^5^ Da) was obtained from Henan Wanbang Industrial Co., Ltd. (Zhengzhou, China). Tween 80 was sourced from Sinopharm Chemical Reagent Co., Ltd. (Shanghai, China). Soy protein isolate was procured from Shandong Yuwang Ecological Food Co., Ltd. (Dezhou, China). Wheat gluten powder was obtained from Fanxian Yellow River Industrial Co., Ltd. (Puyang, China). Hydrochloric acid, sodium hydroxide, methanol, glacial acetic acid, and coomassie brilliant blue R-250 were obtained from Beijing Dingguo Changsheng Biotechnology Co., Ltd. (Beijing, China). Thiobarbituric acid and disodium ethylenediaminetetraacetate were procured from Shenyang Dongxing Reagent Factory (Shenyang, China). Peptone, agar powder, yeast extract, crystal violet neutral red bile salt agar, and brilliant green lactose bile broth were purchased from Beijing Aoboxing Bio-Tech Co., Ltd. (Beijing, China). The *Staphylococcus aureus* chromogenic medium was acquired from Guangdong Huankai Microbial Sci. & Tech. Co., Ltd. (Guangzhou, China). The *Salmonella* chromogenic medium was obtained from Qingdao Haibo Biotechnology Co., Ltd. (Qingdao, China), and vacuum bags were obtained from Shijiazhuang Xilong Packaging Co., Ltd. (Shijiazhuang, China).

### 2.2. Preparation of Essential Oil Nanoemulsion

By modifying Abdalla’s technique slightly, 1.5 g of chitosan powder was dissolved in 100 mL of 1% glacial acetic acid solution and agitated at 60 °C for 30 min, causing a uniform chitosan solution [26]. The solution was mixed with 50 mL of each essential oil at a ratio of 2:1, and 0.2 g (0.18 mL) of Tween 80 was added. The mixture was magnetically stirred at an optimal speed for 30 min, followed by homogenization using a high-speed homogenizer (T25, IKA Instruments Ltd., Kulmbach, Germany) at 15,000 rpm for 3 min, resulting in the formation of a nanoemulsion of LCEO. CEO nanoemulsion was prepared using the same process. A composite essential oil nanoemulsion was prepared by mixing 90 mL of chitosan solution with 30 mL of *Litsea cubeba* oil and 30 mL of cinnamon oil, along with 0.18 mL of Tween 80. After magnetic stirring for 30 min, the mixture was homogenized at 15,000 rpm for 3 min to form the composite essential oil nanoemulsion.

### 2.3. Particle Size, PDI, and Zeta Potential Measurement

Su’s method was modified to accurately measure the particle size, PDI, and zeta potential of essential oil-in-water nanoemulsions [27]. This modification aimed to enhance the precision and reliability of the measurements. Using a nanoemulsion particle size analyzer (ZS90, Malvern Instruments Ltd., Worcestershire, UK), the nanoemulsions were diluted by a factor of 10 before measurement. About 1 mL of the diluted sample was placed in the sample chamber of the instrument, with parameters set as follows: refractive index = 1.70, laser wavelength = 633 nm, and equilibration duration = 120 s.

### 2.4. Stability Evaluation of Essential Oil Nanoemulsion

#### 2.4.1. Measurement of pH Stability

The pH of the essential oil nanoemulsion was calibrated to 3, 5, 7, 9, and 11 with diluted solutions of hydrochloric acid or sodium hydroxide using an improved version of Kang’s methods [28]. The nanoemulsions were left undisturbed for 24 h, after which their stability was evaluated by observing visual appearance, including signs of phase separation, creaming, or flocculation. Further evaluations were conducted to examine the pH stability of the nanoemulsions of the essential oil by analyzing particle size distribution and zeta potential.

#### 2.4.2. Measurement of Freeze–Thaw Stability

After a slight modification to Xiong’s procedure to suit the experimental conditions, the nanoemulsion underwent three freeze–thaw cycles [29]. It was frozen at a temperature of −20 °C for 22 h and thawed in a water bath at a temperature of 40 °C for 2 h. After undergoing freeze–thaw cycles, the nanoemulsion state was evaluated, and the stability was determined by analyzing the particle size and zeta potential.

#### 2.4.3. Measurement of Storage Stability

The generated nanoemulsion containing essential oil was stored at a temperature of 4 °C. The stability of the nanoemulsion was assessed at several time points (0, 60, 120, and 130 days) to determine its long-term storage stability by observing visual appearance, including signs of phase separation, creaming, or flocculation. This procedure provides a thorough evaluation of the long-term stability of the nanoemulsion.

### 2.5. Preparation and Treatment of PBMAs

In this study, a twin-screw extruder (36–40, Hunan Chuangxiang Intelligent Technology Co., Ltd, Changsha, China) was used for the extrusion and cooling molding of PBMAs, using 80% soy protein isolate and 20% wheat gluten as raw materials. The extrusion parameters comprised a temperature of 160 °C, a screw speed of 280 rpm, a solid addition rate of 8.5 kg/h, and a water addition rate of 13.5 kg/h.

Within a controlled and germ-free setting, implements, such as knives and cutting boards, were thoroughly disinfected using a solution containing 75% alcohol. The plant-based meat was cut into pieces of approximately 3 × 3 × 1 cm³ (10 ± 1 g). The pieces were soaked in different diluted essential oils (non-nanoemulsified) and their nanoemulsions for 3 min for preservation. The ratio of plant-based meat mass to essential oil or nanoemulsion volume was maintained at 1:2 (*w*/*v*); specifically, 10 g of meat sample was immersed in 20 mL of essential oil or nanoemulsion. After removing the liquid, the drained samples were then packed in a vacuum bag, hermetically sealed, and placed in a refrigerator (BCD-201E/A, Hisense Ronsheng Refrigerator Co., Ltd, Foshan, China) set at a temperature of 4 °C. The quality indicators of plant-based meat were evaluated every 2 weeks to track temporal variations. This study involved seven treatment groups to evaluate the effectiveness of different methods for preserving plant-based artificial meat. The groups included the control group (CK), LCEO, CEO, combined essential oil (LC/CEO), *Litsea cubeba* essential oil nanoemulsion (LCEON), cinnamon essential oil nanoemulsion (CEON), and combined essential oil nanoemulsion (LC/CEON).

### 2.6. Basic Qualities of PBMAs After Treatment

#### 2.6.1. Color Measurement

Zhou’s method was slightly modified to evaluate the samples using a calibrated colorimeter (CR-400, Konica Minolta Holdings, Inc., Tokyo, Japan) immediately after removal from the refrigerator (BCD-201E/A, Hisense Ronsheng Refrigerator Co., Ltd, Foshan, China) [30]. The luminance (L*) value, chroma (a*) value representing the red–green axis, and chroma (b*) value representing the yellow–blue axis of the samples were recorded. The sample was evaluated thrice, and the average of these evaluations was recorded as the outcome.

#### 2.6.2. pH Measurement

By making slight modifications to the technique of Liang et al. for pH determination, we used a homogenizer to thoroughly mix 5 g of the sample and suspended it in 45 mL of distilled water [31]. The pH of the mixture was determined at the ambient temperature using a pH meter (pHS-25; Shanghai Yidian Scientific Instrument Co., Ltd, Shanghai, China).

#### 2.6.3. Cooking Loss Rate Determination

Adapting Wang’s approach to fit the experimental conditions, the sample was first separated from its packaging, and surplus surface moistures were eliminated using filter paper [32]. The sample mass, denoted as M_1_, was measured after placing it in a cooking bag. The bag was degassed, sealed, and submerged in a water bath at a temperature of 85 °C for 30 min. It was allowed to cool to room temperature, and its mass, denoted as M_2_, was measured. The cooking loss rate was calculated using the following formula:

(1) $$\mathrm{Cooking}\;\mathrm{Loss}\;\mathrm{Rate}\;\mathrm{Determination}\;(\%)=\frac{M_{1}\;-M_{2}}{M_{1}}\times 100\%$$

#### 2.6.4. Lipid Oxidation Using TBARS Analysis

Using an improved version of the technique developed by Dai et al. (2022), 5 g of plant-based synthetic meat was added to 50 mL of trichloroacetic acid at a concentration of 0.5 M [11]. The solution was stirred at a temperature of 50 °C for 30 min and cooled to the ambient temperature. After filtering, 5 mL of the resulting liquid was mixed with 5 mL of a solution containing 0.02 M thiobarbituric acid. The solution was placed in a water bath at a temperature of 90 °C for 30 min. After the solution was cooled, the absorbance was measured at 532 nm using a UV-visible spectrophotometer (UV-8000A, Shanghai Instrument & Electrical Equipment Co., Ltd., Shanghai, China). The TBARS value was determined by measuring the amount of malondialdehyde in the sample and was expressed in mg MDA/kg.

#### 2.6.5. Total Viable Count (TVC) Determination

Using Weil’s technique, a 25 g portion of the sample was measured and placed in a stomacher bag along with 225 mL of a saline solution with a concentration of 0.85 g/L [33]. The mixture was blended for 3 min. Subsequently, the standardized sample was diluted at a ratio of 1:10 using a saline solution with a concentration of 0.85 g/L. Three Petri dishes were inoculated with 1 mL of the final dilution, each in triplicate. The plates were placed in an incubator (JQ-1000-TF, Dongguan Qingsheng Testing Equipment Co., Ltd, Dongguan, China) set at a temperature of 37 °C and left undisturbed for 48 h.

#### 2.6.6. *Escherichia coli*/*Staphylococcus aureus* Count Determination

Using the diluted solution made earlier, 1 mL of the mixture was placed on violet red bile agar and kept in an incubator at a temperature of 37 °C. After 24 h, plates with 15–150 suspicious *E. coli* colonies were chosen. Ten colonies were inoculated into 10 test tubes with brilliant green lactose bile broth to observe gas generation and record the number of tubes showing the presence of gas. The product of the gas-positive tube ratio and the number of counted plate colonies provided the *E. coli* count/g of the sample. To measure the amount of *S. aureus*, a 1 mL sample of the liquid was placed on mannitol salt agar and allowed to grow for 24 h. The number of red colonies was recorded.

#### 2.6.7. Fourier Transform Infrared Spectroscopy (FTIR)

Using Peng’s approach as a basis, plant-based imitation meat samples were treated with six preservatives and stored for different durations [34]. Additionally, a control group without preservatives was included. Samples were freeze-dried (Scientz-12N; Ningbo Xinzhi Biotechnology Co., Ltd, Ningbo, China). The obtained powder was compacted against a flat sample stage using a metal pin in the ATR setup. The spectral scan range was set from 4000 to 400 cm^−1^, with an instrument resolution of 4 cm^−1^ and a wavenumber precision of 0.01 cm^−1^.

### 2.7. Sensory Evaluation

The sensory panel consisted of 10 trained individuals aged between 22 and 26 years, who are currently graduate students at the College of Grain Science and Technology, Shenyang Normal University, China. They were fully informed about the purpose of this study and provided written consent to participate in the experiments. The study protocol was approved by the Research Ethics Committee of the College of Grain Science and Technology, ensuring compliance with ethical standards for human research. Prior to the sensory evaluation, the panel members underwent a training course. The scoring rules are listed in Table 1. The plant-based meat was removed from the refrigerator. The outer packaging of the test samples was opened and positioned on the sensory scoring operation table in a certain order. The sensory panel members were restricted from having or coming into contact with stimulating foods or goods before performing an assessment. They evaluated the plant-based meat according to their preferences and sensory rating sheets.

### 2.8. Statistical Analysis

The experiments were performed thrice, and the results were analyzed using one-way ANOVA and Duncan’s multiple range test in SPSS 26.0. A *p*-value < 0.05 was considered to have statistical significance, and the findings were presented as the mean value plus or minus the standard deviation.

## 3. Results and Discussion

### 3.1. Characterization of Essential Oil Nanoemulsion

The size of the particles in an emulsion system is a crucial component that affects its stability and dispersion capacity. This impacts the solubility and bioavailability of hydrophobic substances inside the dispersed phase of a nanoemulsion [35]. The particle sizes for LCEON, CEON, and LC/CEON were 7.08 ± 0.07, 6.62 ± 0.29, and 4.99 ± 0.03 nm, respectively (Table 2). The results indicated that the LC/CEON emulsion exhibited the smallest droplet size, suggesting a more stable and well-dispersed system than the other samples. This may be attributed to the particular interactions between the lipid components and the emulsifier in the LC/CEON formulation, which may cause improved packing and stability of the oil droplets [36].

PDI is a pivotal metric in assessing the stability and uniformity of emulsion systems, with lower PDI values indicating a narrow size distribution and enhanced stability [37]. The results showed the PDI values for LCEON (0.228 ± 0.003), CEON (0.454 ± 0.016), and LC/CEON (0.221 ± 0.008). This indicates that LCEON and LC/CEON had a more consistent size distribution than CEON (Table 2). The difference in the PDI values may be attributed to the diverse molecular configurations and interactions inside the emulsions, which affect the consistency of the droplet sizes. Specifically, the molecular structure and hydrophobicity of the essential oils, as well as the interactions between the oil droplets and the chitosan–Tween 80 complex, can influence the droplet size distribution and stability of the emulsions. The PDI values for all formulations were below 0.5, suggesting a consistent and even droplet dispersion [38].

The zeta potential is a crucial factor for assessing the stability and dispersibility of nanoemulsions. Higher absolute values indicate a more stable system owing to enhanced electrostatic repulsion between particles [39]. The zeta potential examination of nanoemulsions revealed that LCEON, CEON, and LC/CEON had zeta potentials of 62.13 ± 2.30, 61.83 ± 1.92, and 95.13 ± 2.67 mV, respectively (Table 2). The significant increase in the zeta potential observed in LC/CEON indicates a higher level of stability in the emulsion. This was possibly owing to the combined action of the essential oils and the adjusted chitosan-to-oil ratio. Notably, there is a correlation between the particle size and zeta potential. For instance, nanoemulsions with smaller particle sizes tend to have higher absolute zeta potential values, which enhances their stability by increasing electrostatic repulsion between droplets. Yin et al. reported that when the zeta potential exceeds +30 mV or falls below −30 mV, there is a significant electrostatic repulsion between the droplets [40]. This repulsion prevents coagulation. Consequently, the increased electrostatic repulsion substantially reduces droplet clustering. LC/CEON demonstrated reduced particle size, a low PDI, increased absolute zeta potential values, and improved system stability.

### 3.2. Stability Evaluation of Essential Oil Nanoemulsion

#### 3.2.1. pH Stability

**Figure 1:**
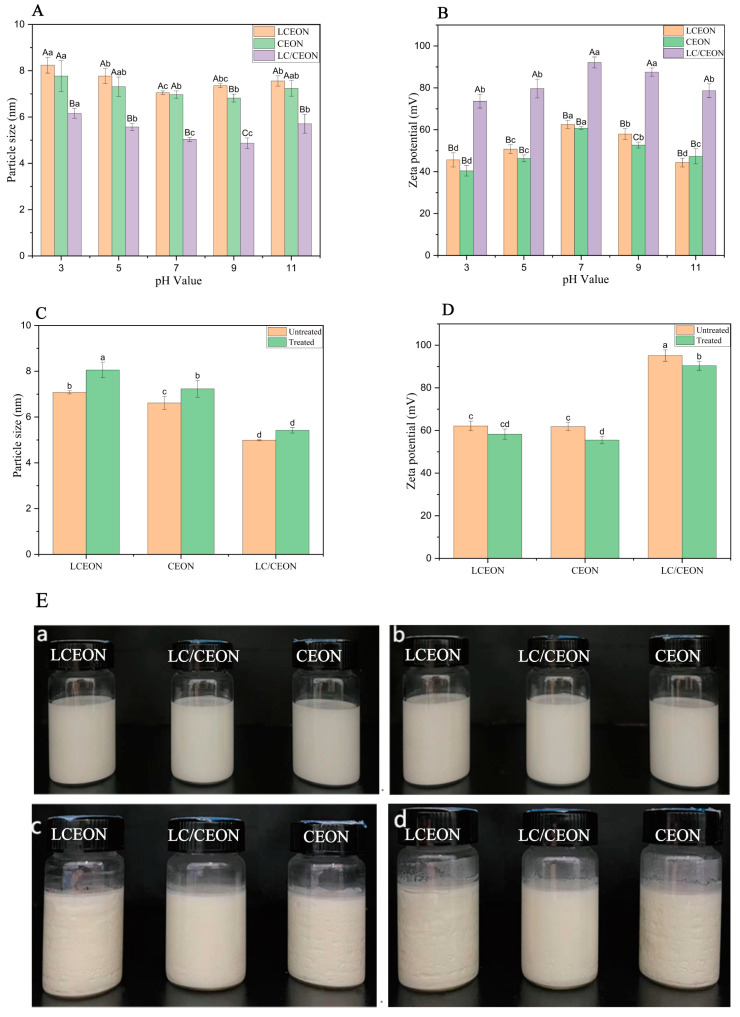
(**A**) Particle size of essential oil nanoemulsion at different pH. (**B**) Zeta potential of essential oil nanoemulsion at different pH. (**C**) Particle size of freeze–thaw treatment of essential oil nanoemulsion. (**D**) Zeta potential of freeze–thaw treatment of essential oil nanoemulsion. (**E**) Appearance pictures of three essential oil nanoemulsions during storage at 4 °C (a: 0 days of storage; b: storage for 60 days; c: storage for 120 days; d: storage for 130 days; the order from left to right is *Litsea cubeba* essential oil nanoemulsion, complex essential oil nanoemulsion, and cinnamon essential oil nanoemulsion). (**A**,**B**) In the same histogram, different uppercase letters indicate that the differences between the essential oil nanoemulsions at the same pH value are statistically significant, while different lowercase letters indicate that the differences in the same essential oil nanoemulsion at different pH values are statistically significant (*p* < 0.05). (**C**,**D**) Different lowercases in the same histogram indicate statistically significant differences (*p* < 0.05).

Figure 1A,B illustrates the relationship between pH and particle size of the nanoemulsions. As the pH decreased from 7 to 3, there was a notable increase in the particle size of the emulsions. This trend can be attributed to the increased concentration of hydrogen ions at lower pH values, which affects the stability of the nanoemulsion system. The droplet size of the essential oil nanoemulsion decreased, and the zeta potential increased at pH values of 7 and 9, indicating that the most uniform droplet dispersion occurred at a neutral pH. In contrast, in the pH range of 9–11, the droplet size increased, and the zeta potential declined. The decrease in pH (Figure 1A,B, *x*-axis) led to an increase in the ionic strength of the solution, causing the double electric layer to compress, resulting in an improved shielding effect of the counterions. This reduced the absolute value of the zeta potential. The shielding effect decreases the charge on the surface of the particles, causing a reduced repulsion between the particles and an increased possibility of agglomeration. Consequently, the droplets become less stable [41]. The three categories of essential oil nanoemulsions showed negligible variations in droplet size and zeta potential over the pH range of 3–11, suggesting excellent pH stability. The stability of the composite essential oil nanoemulsions is possibly owing to the combined action of chitosan and Tween 80 as emulsifiers. These nanoemulsions show excellent pH stability.

#### 3.2.2. Freeze–Thaw Stability

There were varying droplet sizes in essential oil nanoemulsions after three freeze–thaw cycles compared with those in the untreated samples (Figure 1C,D). However, the droplet size variations in the three forms of essential oil nanoemulsions were limited to 1 nm. The greatest change was recorded in the LCEO nanoemulsion, showing a post-treatment rise of 0.98 nm. This result is consistent with that of Hou et al., where these phenomena are attributed to crystallization-induced physical and chemical changes [42]. At the beginning of the thawing process, the frozen emulsion caused the interfacial layer to break, prompting the merging of droplets and the separation of oil and water. Consequently, when heated, the higher speed at which molecules move increases the possibility of collisions between emulsion particles [43]. This caused smaller particles to combine and form larger particles, resulting in an increase in the droplet size. Following three freeze–thaw cycles, the zeta potential variations in the three nanoemulsions containing essential oils were notably large, particularly for CEO nanoemulsions. This phenomenon arises from the separation of some chemicals from the droplet membrane during the freeze–thaw process, causing a decrease in zeta potential [44]; however, nanoemulsions with thicker interfacial layers can generate stronger repulsive forces on the droplet surfaces. This makes it more challenging for ice crystals to enter a thicker interfacial film, causing reduced coalescence and improved freeze–thaw stability. The three categories of essential oil nanoemulsions demonstrated excellent freeze–thaw stability.

#### 3.2.3. Storage Stability

There was no creaming or phase separation in the newly made essential oil emulsions and those held for 60 days at 4 °C (Figure 1E(b)). The appearance of the emulsions remained similar during the storage period, demonstrating the stability of the three types of essential oil nanoemulsions. On the 120th day (Figure 1E(c)), visible solid particles were observed in the LCEO and CEO nanoemulsions, indicating decreased stability; however, the composite essential oil remained stable. On the 130th day, a significant quantity of solid particles was formed in the LCEO and CEO nanoemulsions, possibly owing to settling or solidification. However, the composite essential oil nanoemulsion contained only a small number of solid particles at the bottom. After being held at a temperature of 4 °C for 130 days, the three types of essential oil nanoemulsions exhibited no signs of stratification or demulsification. The experimental findings demonstrate the stability of the three types of essential oil nanoemulsions generated using the aforementioned procedure.

### 3.3. Color Analysis

Table 3 presents the alterations in the lightness (L*), redness (a*), and yellowness (b*) of PBMAs treated with various essential oil nanoemulsions. The luminosity of PBMA surfaces depends on the level of moisture and the specific proteins produced [45]. On Day 0, the visual appearance of all PBMA samples was uniform, with a light brown color typical of plant-based meat alternatives. Empirical investigations showed that as the storage duration increased, the overall brightness values of all specimens diminished progressively, with notable decreases in the L* values (*p* < 0.05). This reduction in lightness can be attributed to several factors: moisture loss, protein degradation, and oxidation of protein [45]. The control group without any treatment showed the most significant and rapid reduction in the L* value. It started at 56.14 and dropped to 45.04 on the 28th day, causing a decrease of 39.64%. On the 28th day, samples treated with *Litsea cubeba* seed oil and CEO showed 10.64% and 8.82% reductions in L* values, respectively. On the 119th day of storage, the L* value of the composite essential oil nanoemulsion was measured at 45.75. This indicated a stronger preservative effect than that of the essential oils alone during the same period. The enhanced preservation is possibly owing to the antibacterial and antioxidant properties of the composite essential oil nanoemulsion, causing slower darkening of the samples [46,47]. However, the L* value of LC/CEON is slightly lower than that of LC/CEO. This may be due to the nanoemulsified essential oil causing a certain degree of turbidity on the sample surface. This turbidity could affect the light reflection and absorption by the colorimeter, resulting in a lower measured L* value.

The control group showed the fastest drop in redness (a*) values, causing the sample surfaces to become dark brown by the 28th day, possibly because of protein oxidation [48]. Owing to the intrinsic darkness of PBMAs, there was no significant change in a* values (*p* < 0.05). However, the essential oil nanoemulsion showed a more effective deceleration of the decrease in a* values of plant-based imitation meat than the samples treated with essential oils alone. Moreover, the composite essential oil treatment was superior to the individual essential oil treatments.

The chroma (b* values) of all samples increased with prolonged storage duration. The group without any treatment showed the most significant increase in growth, followed by the group treated with a single essential oil and the group treated with a combination of essential oils, which had identical growth rates. The group treated with a nanoemulsion of the combination essential oil had the lowest growth rate. The increase in b* values is associated with increased lipid oxidation, and the increase in meat TBARS levels in this study further aggravated the increase in b* values [49]. The analysis of the three color parameters suggests that the essential oil nanoemulsion effectively preserved PBMAs because of its exceptional ability to retain water, its antioxidant capabilities, and its antibacterial activity.

### 3.4. pH Value

**Figure 2:**
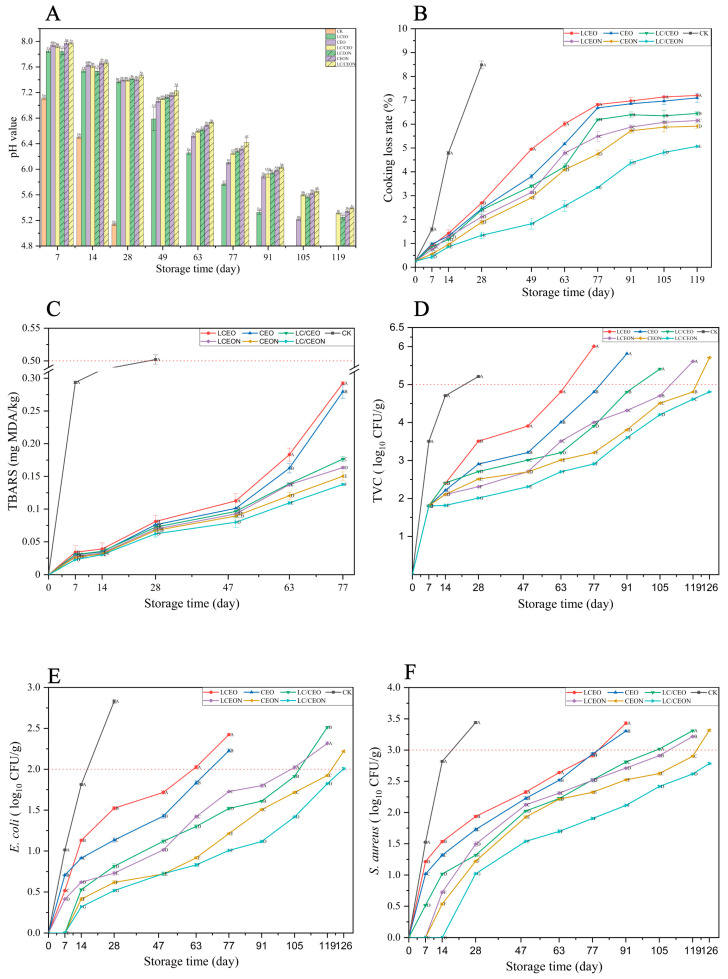
(**A**) Effect of essential oil nanoemulsion on pH of plant-based meat analogs. (**B**) Effect of essential oil nanoemulsion on the cooking loss rate of plant-based meat analogs. (**C**) Effect of essential oil nanoemulsion on thiobarbituric acid reactive substances of plant-based meat analogs. (**D**) Effect of essential oil nanoemulsion on the total viable count (TVC) of plant-based meat analogs. (**E**) Effect of essential oil nanoemulsion on *Escherichia coli* of plant-based meat analogs. (**F**) Effect of essential oil nanoemulsion on the number of *Staphylococcus aureus* of plant-based meat analogs. Different uppercase letters indicate that the differences between the essential oil nanoemulsions at the same storage days are statistically significant, while different lowercase letters indicate that the differences in the same essential oil nanoemulsion at different storage days are statistically significant (*p* < 0.05).

All samples showed a notable decline in pH with increasing storage time (*p* < 0.05) (Figure 2A). This observation aligns with the results of Dai et al., who also observed a similar pH trend in PBMAs [11]. The pH reduction may be caused by the production of lactic acid by yeast and lactic acid bacteria or by the oxidation of vitamins [50,51]. The untreated control group showed the most significant and rapid decrease, with the pH decreasing from 7.10 on Day 7 to 5.13 after 28 days of storage. Compared with the control group, the group treated with essential oil exhibited a decelerated decrease in pH, particularly following the formation of nanoemulsions; the pH reduction was even more gradual. During the analysis, the samples treated with composite essential oil nanoemulsions consistently exhibited elevated pH levels compared with those in the other groups. Significantly, the use of essential oils consistently prevented a decreased pH until Day 119. Plant-based meat substitutes treated with nanoemulsions of essential oils maintain a high level of freshness. This is particularly true for plant-based meat substitutes treated with nanoemulsions of composite essential oils. This suggests that composite essential oil nanoemulsions have a strong preservation effect on plant-based meat substitutes.

### 3.5. Cooking Loss

Cooking loss refers to the ability of the meat to retain water at high temperatures, and it is a significant factor that affects meat juiciness [52]. The lost liquid primarily comprises water; however, it also includes soluble proteins, inorganic salts, vitamins, and other extracts [53]. The cooking loss rate of PBMAs was usually increased when they were refrigerated (Figure 2B). During extrusion molding, the cooking loss rate was 0.25%, while the control group showed a cooking loss rate of 8.5% on the 28th day. The concentration of the essential oil extracted from *Litsea cubeba* was 7.20% on the 119th day, whereas the nanoemulsion of LCEO showed a cooking loss rate of 6.15% on the same day. Significantly, the samples treated with nanoemulsions of the composite essential oil exhibited the least rapid increase in the rate of cooking loss, reaching 5.06% by the 119th day. These findings indicate that the nanoemulsions successfully inhibited moisture loss in meat samples, most possibly because of their compact structure and robust water barrier characteristics, effectively retaining moisture. Consistent with these observations, the cooking loss rate of PBMAs during storage was inversely correlated with the pH levels. The primary cause of this is lactic acid, which is formed by yeast and lactic acid bacteria, and acidic chemicals produced by microorganisms that produce acid during their metabolic processes. These factors cause a reduction in the pH of the sample [54]. At the isoelectric point, proteins have a net charge of zero because the pH reaches its limit. This causes protein–protein attraction and a decrease in their affinity for water, resulting in reduced water retention [54]. The use of essential oil nanoemulsions for preserving PBMAs may cause a reduced cooking loss rate and improved sales during a 3-month timeframe.

### 3.6. TBARS Value

The TBARS values of PBMAs treated with the essential oil and essential oil nanoemulsions initially increased and decreased (Figure 2C). This tendency is similar to the pattern of lipid oxidation in beef observed by Khan et al., where a decrease in TBARS values may be owing to the formation of tertiary lipid oxidation products [55]. In 28 days, the TBARS values of the untreated control group increased rapidly, reaching a threshold on the 28th day, with values >0.5 mg/kg. This caused the development of an unpleasant odor, indicating severe lipid oxidation in the samples [56]. Similarly, samples treated with essential oil showed relatively high levels of lipid oxidation. The TBARS values reached 0.3 mg/kg by the 77th day. This could be attributed to the limited ability of the essential oil to block oxygen, as the high oxygen content in PBMAs promotes oxidation [57]. Conversely, when essential oil nanoemulsions were used to package PBMAs, the TBARS values were substantially decreased, reaching 0.14 mg/kg by the 77th day. Furthermore, the use of composite essential oil nanoemulsions was more efficient in preventing lipid oxidation in PBMAs than the use of essential oil nanoemulsions alone (*p* < 0.05). These findings align well with the superior performance observed in essential oil nanoemulsions, particularly composite ones, in preventing lipid oxidation. Nanoemulsions provide a more uniform and stable dispersion of essential oils throughout the PBMA matrix, ensuring better coverage and prolonged antioxidant activity. The nano-sized droplets of essential oils in nanoemulsions have a significantly larger surface area-to-volume ratio compared to bulk essential oils, allowing for more efficient interaction with free radicals and other oxidizing agents [58]. In composite essential oil nanoemulsions, the combination of different essential oils may lead to synergistic antioxidant effects, where the combined action is greater than the sum of individual components [59]. Additionally, nanoemulsions can provide a controlled release of antioxidant compounds over time, maintaining their effectiveness throughout the storage period. The small size of nanoemulsion droplets may also allow for better penetration into the meat matrix, providing antioxidant protection at a molecular level [60]. These factors collectively contribute to the enhanced antioxidant efficacy of essential oil nanoemulsions, particularly composite ones, in preventing lipid oxidation in PBMAs. Further research is needed to elucidate the specific mechanisms and optimize the composition of these nanoemulsions for maximum antioxidant performance in meat preservation.

### 3.7. TVC Value

The total viable count (TVC) grew as storage time progressed (Figure 2D). This can be attributed to the high protein content of the samples, which provided a favorable environment for microbial development. If the total viable count exceeds 5.0 log_10_CFU/g, it indicates spoilage, which means that the sample is not suitable for consumption [61]. The untreated control group attained a bacterial count of 5.21 log_10_CFU/g on the 28th day of storage, confirmed by the observed change in the cooking loss rate during storage. On the 77th, 91st, and 105th days, the number of samples treated with LCEO, CEO, and the composite essential oils, respectively, exceeded the threshold. In contrast, the samples exposed to the nanoemulsions of these specific essential oils had a bacterial count of >5.0 log_10_CFU/g after storage for 119 and 126 days. Samples treated with the composite essential oil nanoemulsion had a total viable count of 4.81 log_10_CFU/g on the 126th day. This count did not exceed the threshold of 5.0 log_10_CFU/g. The effective antimicrobial properties of the essential oil nanoemulsions inhibited the growth of most microorganisms [62].

### 3.8. Microbiological Analysis

Figure 2E shows the effects of several treatments on the levels of *E. coli* in PBMAs. Over time, the number of *E. coli* cells steadily increased, with the control group showing the most rapid growth. After 28 days of storage, the *E. coli* count in the control group reached 2.83 log_10_CFU/g, exceeding the recommended limit of 2.00 log_10_CFU/g set by the Group Standard issued by Chinese Institute of Food Science and Technology (T/CIFST 001-2020) [63]. On the 105th day, the samples treated with LCEO, CEO, and LCEO nanoemulsion had *E. coli* counts exceeding 2.00 log_10_CFU/g. However, the samples treated with the mixture of the two essential oils had bacterial counts approaching 1.91 log_10_CFU/g, which was close to the threshold. Significantly, after 7 days of storage, PBMAs treated with the composite essential oil, CEO, and composite essential oil nanoemulsions exhibited no measurable presence of *E. coli*. The essential oils altered the structure and permeability of bacterial cell membranes. This caused the leakage of small electrolytes and the release of proteins and nucleic acids. Consequently, the integrity of the cell membrane was compromised, causing a decrease in bacterial metabolic activity [20]. The application of nanoemulsions containing composite essential oils increased the longevity of the samples to 119 days while maintaining *E. coli* levels below 2.00 log_10_CFU/g. The enhanced distribution and release of essential oils by nanoemulsions caused increased durability and stability of their antibacterial properties [64]. Composite essential oil nanoemulsions showed exceptional effectiveness in suppressing the growth of *E. coli* in PBMAs.

Consistent with the pattern shown in Figure 2F, the number of *S. aureus* increased as the storage duration increased. After storage for 28 days, the count of *S. aureus* in the blank control sample reached 3.44 log_10_CFU/g, which exceeds the recommended limit of 3.00 log_10_CFU/g as per the Group Standard issued by Chinese Institute of Food Science and Technology (T/CIFST 001-2020) [63]. On the 91st day of storage, the number of *S. aureus* in LCEO and CEO exceeded the permitted range, whereas those in the other treated samples remained below acceptable limits. According to Zhang et al., *S. aureus* is more susceptible than *E. coli* to CEO. The graph demonstrates that the composite essential oil nanoemulsion is highly efficient in suppressing the growth of *S. aureus* [20]. On the 14th day of the experiment, no traces of *S. aureus* were detected in meat samples treated with the nanoemulsion. There was a notable correlation between the duration of storage and the growth of *S. aureus* (*p* < 0.05) under various treatment conditions. Composite essential oil nanoemulsions have superior performance compared with nanoemulsions containing a single essential oil, which is more successful than treatments using composite essential oils. Notably, LCEO demonstrated the least preservative activity. The experimental findings demonstrate that nanoemulsions of essential oils successfully suppress *S. aureus* growth in PBMAs.

### 3.9. FTIR Analysis

The secondary structure of proteins is distinguished by different hydrogen bonds, leading to distinct absorption peaks in the amide I band. The primary secondary structures of proteins include α-helix, β-sheet, β-turn, and random coil [65]. The amide I peak assignments for these structures are as follows: α-helix (1650–1660 cm^−1^), β-sheet (1600–1640 cm^−1^), β-turn (1660–1700 cm^−1^), and random coil (1640–1650 cm^−1^) [66]. Protein oxidation, which occurs during the storage of PBMAs, results in a decrease in α-helix content and an increase in β-turns and random coils. These changes are considered important indicators for evaluating protein oxidation [67].

**Figure 3:**
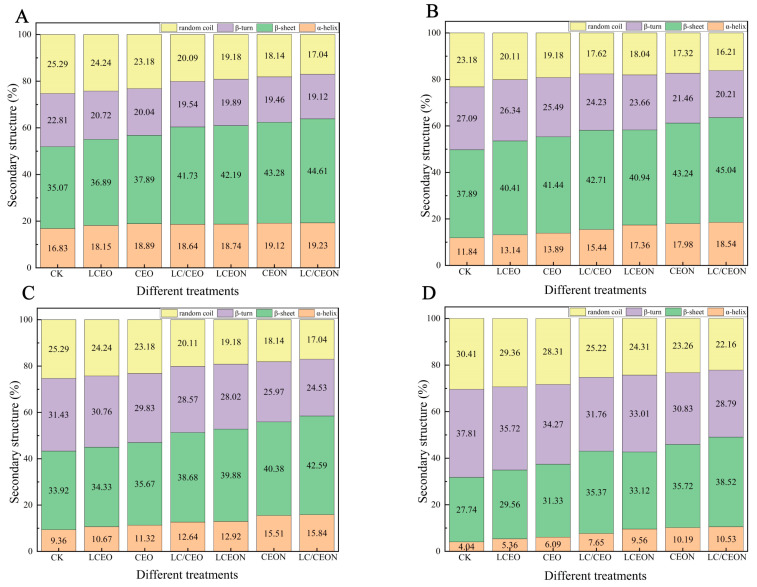
Effects of nanoemulsion treatment with different essential oils on the secondary structure of plant-based meat in protein at different days ((**A**): the secondary structure diagram of protein; (**B**): the secondary structure diagram of protein; (**C**): the secondary structure diagram of protein; (**D**): the secondary structure diagram of protein).

As shown in Figure 3, the α-helix content significantly dropped as the storage duration increased, whereas the content of β-turns increased. In the control group without any treatment, the proportion of α-helix decreased by 5.0% on Day 35 compared with Day 7, while the proportion of β-turns increased by 4.29%. On Day 84, the proportion of α-helix decreased by 2.48% compared with Day 35, and the proportion of β-turns increased by 4.34%. Finally, on Day 105, the proportion of α-helix decreased by 5.31% compared with Day 84, and the proportion of β-turns increased by 6.37%. These changes indicate that the secondary structure of proteins in the samples was disrupted, causing structural disorder and confirming protein oxidation during storage [68]. Additionally, PBMAs that were not treated had a relative α-helix content of 16.84% and a β-turn content of 22.8% after 7 days of storage. Compared with the control group, samples treated with LCEO showed a 1.31% increase in α-helix content and a 2.08% decrease in β-turn content. Similarly, samples treated with CEO showed a 2.05% increase in α-helix content and a 2.40% decrease in β-turn content. Furthermore, the disparity between the two groups grew larger. On Day 105, the β-turn content of CEO was 3.53% lower than that in the control group. This indicates that treatment with a single essential oil can hinder protein oxidation in PBMAs. This effect is attributed to the presence of phenolic compounds in the essential oils, which can impede the conversion of α-helix and preserve the protein’s secondary structure [69]. In addition, samples treated with composite essential oil exhibited a greater proportion of α-helix than those treated with individual essential oils. The relative content of irregular curls was lower in the samples treated with composite essential oils, suggesting that the anti-protein oxidation effect of composite essential oils was superior to that of the individual essential oils. When essential oils were turned into nanoemulsions and applied to PBMAs, the α-helix content in both single essential oils was higher than the essential oil-treated samples, at 0.59% and 0.23%. The β-turns content was lower in the nanoemulsions, at 0.83% and 0.58%, indicating that essential oil nanoemulsions have a greater ability to prevent protein oxidation in PBMAs than essential oils alone. The use of composite essential oil nanoemulsions surpassed all other treatments and exhibited the highest efficacy in preventing protein oxidation in PBMAs.

### 3.10. Sensory Evaluation

**Figure 4:**
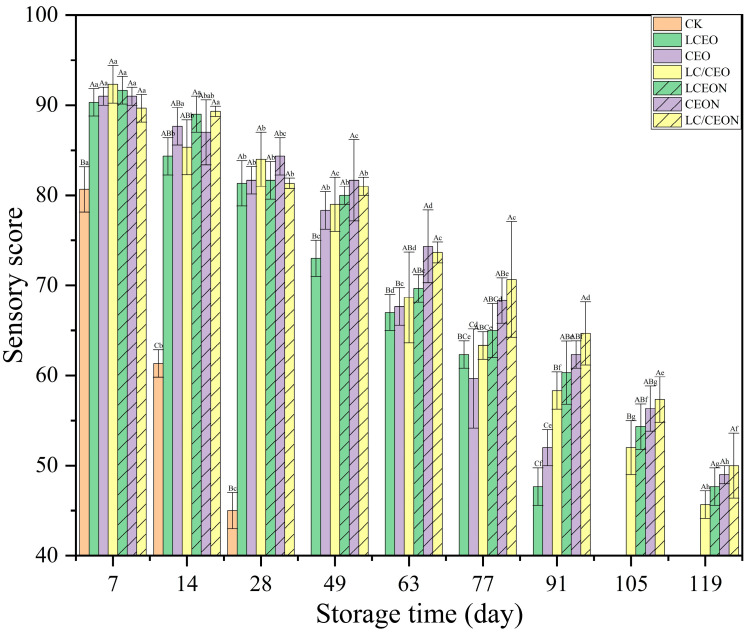
Effect of essential oil nanoemulsion on the sensory scores of plant-based meat analogs. Different uppercase letters indicate that the differences between the essential oil nanoemulsions at the same storage days are statistically significant, while different lowercase letters indicate that the differences in the same essential oil nanoemulsion at different storage days are statistically significant (*p* < 0.05).

Sensory analysis was used to evaluate PBMAs using the visual, tactile, and olfactory senses. It was used to measure factors such as color, odor, and muscle elasticity after storage (Figure 4). As shown in Figure 4, the sensory scores of all samples gradually decreased with increasing storage time. On the 28th day, the sensory indications of the control group were considered unsatisfactory, as the sensory scores decreased to 45 points, meat color became darker, and muscle texture suppleness declined. When LCEO, CEO, or a combination of both was used, the samples showed a slower decrease in sensory scores than the control group. In sensory evaluation, the use of LCEO and CEO extended the storage duration of the samples to 91 days. Furthermore, the combined treatment of essential oils maintained the overall acceptability of the samples until Day 105, with a sensory evaluation score of 52 points. Over time, the samples treated with essential oil nanoemulsions had a more gradual decrease in sensory ratings than the group treated with essential oil. Specifically, the sensory evaluation of plant-based imitation meat treated with a combination of essential oil nanoemulsion resulted in a score of 50 points on Day 119. As the storage period increased, the sensory scores for the various treatments significantly decreased (*p* < 0.05). The combined essential oil nanoemulsion performed better than the single essential oil nanoemulsions, which performed better than the combined essential oil treatment. Among the essential oils, LCEO exhibited the least effective preservation effect. Off-flavors and discoloration in plant-based imitation meat can be attributed to the growth of microorganisms, breakdown of proteins, and oxidation of lipids. The experimental findings indicate that nanoemulsions of essential oils successfully delay the degradation of the sensory attributes of PBMAs.

## 4. Conclusions

In this study, chitosan and Tween 80 were used as emulsifying ingredients to create nanoemulsions of LCEO and CEO using a high-pressure homogenization technique. The findings indicated that the composite essential oil nanoemulsion exhibited its best characteristics when the chitosan solution was mixed with LCEO and CEO in a ratio of 3:2, with equal amounts of each oil and 0.2 g of Tween 80. The composite essential oil nanoemulsion displayed a smaller particle size, lower PDI, higher absolute zeta potential, and improved emulsion stability than the previous emulsions. Subsequently, the selected nanoemulsion was used to protect plant-based artificial meat at a temperature of 4 °C. The findings indicate that the samples treated with the composite essential oil nanoemulsion showed substantial enhancements in color, pH, TBARS, and cooking loss rate compared with the control and other groups. The addition of the composite essential oil nanoemulsion to plant-based artificial meat reduced the growth of spoilage bacteria, particularly showing stronger inhibition against *E. coli* and *S. aureus*. Similarly, it reduced protein oxidation in meat, prolonging the shelf life of plant-based artificial meat and delaying the deterioration of its sensory properties. Therefore, in the preservation process of PBMAs, the encapsulation of LCEO/CEO is capable of effectively inhibiting the spoilage and deterioration of PBMAs. Based on the experimental results, compared to PBMAs without any additives, those treated with LCEO/CEO nanoemulsion showed an extended storage time of 98 additional days. This significant increase in storage time demonstrates that incorporating nanoemulsified LCEO/CEO is more effective in suppressing the spoilage of PBMAs than the direct addition of LCEO/CEO, thus leading to improved flavor and quality of PBMAs. In the future, a broader range of essential oils could be emulsified into nanoemulsions by various emulsifiers to inhibit the spoilage of PBMAs during storage. Furthermore, researchers might investigate alternative encapsulation techniques to encapsulate plant essential oils to prevent the deterioration of PBMAs, such as microcapsule and nanoparticle methods.

## Figures and Tables

**Table 1:** Sensory evaluation table.

Score Metrics	Specific Rules	Scores
Color (20 points)	Pale yellow, shiny, and mildew-free	13–20 points
	Pale yellow, with lost luster and mildew-free	7–12 points
	Grayish or darker yellow with mildew	0–6 points
Form (35 points)	Great elasticity, moderate hardness, with obvious meat fiber, no mucus	27–35 points
	Good elasticity, too hard or too soft, without obvious meat fiber, no mucus	14–16 points
	Poor elasticity, unacceptable hardness, no meat fiber, mucus	0–13 points
Odor (35 points)	Strong fragrance of specific products, no odor, no acidic taste	27–35 points
	Fragrance of a specific product, no odor, no acidic taste	14–16 points
	Weak fiber, smelly, sour	0–13 points
Impurities (10 points)	No impurities visible to the naked eye	10 points

**Table 2:** Particle size, PDI, and zeta potential of essential oil nanoemulsion.

Nanoemulsion	Particle Size (nm)	PDI	Zeta Potential (mV)
LCEON	7.08 ± 0.07 ^a^	0.228 ± 0.003 ^b^	62.13 ± 2.30 ^b^
CEON	6.62 ± 0.29 ^b^	0.454 ± 0.016 ^a^	61.83 ± 1.92 ^b^
LC/CEON	4.99 ± 0.03 ^c^	0.221 ± 0.008 ^b^	95.13 ± 2.67 ^a^

LCEON, *Litsea cubeba* essential oil nanoemulsion; CEON, cinnamon essential oil nanoemulsion; LC/CEON, a combination of *Litsea cubeba* and cinnamon essential oil nanoemulsions; PDI, polydispersity index. Data are presented as means ± standard deviation. Different letters in the same column indicate significant differences (*p* < 0.05).

**Table 3:** Changes in lightness (L*), redness (a*), and yellowness (b*) values of PBMAs treated with different essential oil nanoemulsions.

Color Parameter	Storage (Day)	CK	LCEO	CEO	LC/CEO	LCEON	CEON	LC/CEON
L*	0	56.14 ± 0.97 ^Aa^	56.14 ± 0.97 ^Aa^	56.14 ± 0.97 ^Aa^	56.14 ± 0.97 ^Aa^	56.14 ± 0.97 ^Aa^	56.14 ± 0.97 ^Aa^	56.14 ± 0.97 ^Aa^
	7	53.28 ± 2.60 ^Aab^	55.74 ± 2.00 ^Aab^	55.55 ± 0.41 ^Aab^	56.01 ± 1.23 ^Aa^	55.37 ± 1.36 ^Aa^	55.67 ± 3.16 ^Aab^	55.37 ± 0.75 ^Aab^
	14	50.17 ± 1.92 ^Cb^	52.10 ± 0.90 ^BCc^	54.62 ± 1.13 ^Aab^	54.23 ± 1.42 ^ABab^	55.67 ± 0.94 ^Aa^	54.95 ± 0.79 ^Aabc^	53.62 ± 1.01 ^ABbc^
	28	45.04 ± 1.20 ^Bc^	53.16 ± 1.32 ^Abc^	53.67 ± 1.26 ^Ab^	55.16 ± 2.01 ^Aa^	54.64 ± 1.78 ^Aab^	53.67 ± 1.91 ^Aabcd^	53.17 ± 1.67 ^Acd^
	49	ND	50.63 ± 1.11 ^Bcd^	50.31 ± 2.06 ^Bc^	53.86 ± 0.97 ^Aab^	52.33 ± 2.29 ^Abc^	52.26 ± 0.85 ^Abcd^	52.06 ± 0.95 ^Acd^
	63	ND	48.33 ± 2.31 ^Ade^	49.42 ± 1.79 ^Acd^	51.74 ± 1.60 ^Abc^	51.97 ± 1.33 ^Abc^	51.79 ± 2.65 ^Acd^	51.12 ± 1.87 ^Ade^
	77	ND	45.69 ± 0.75 ^Cef^	47.54 ± 1.56 ^BCd^	50.64 ± 0.99 ^Acd^	50.10 ± 1.64 ^ABcd^	50.97 ± 2.12 ^Ade^	49.79 ± 1.16 ^ABef^
	91	ND	44.50 ± 1.60 ^Cf^	45.34 ± 0.94 ^BCe^	48.58 ± 2.34 ^Ade^	48.87 ± 0.97 ^Ade^	48.33 ± 1.64 ^ABef^	48.31 ± 2.03 ^ABfg^
	105	ND	43.76 ± 1.57 ^Cf^	44.22 ± 1.31 ^BCe^	47.39 ± 1.68 ^Ae^	46.63 ± 2.03 ^ABef^	45.61 ± 1.33 ^ABCfg^	46.35 ± 0.18 ^ABCgh^
	119	ND	43.01 ± 2.46 ^Bf^	43.64 ± 0.76 ^ABe^	46.59 ± 1.15 ^Ae^	45.39 ± 1.96 ^ABf^	43.26 ± 1.94 ^Bg^	45.75 ± 0.16 ^ABh^
a*	0	6.23 ± 0.75 ^Aa^	6.23 ± 0.75 ^Aa^	6.23 ± 0.75 ^Aa^	6.23 ± 0.75 ^Aa^	6.23 ± 0.75 ^Aa^	6.23 ± 0.75 ^Aa^	6.23 ± 0.75 ^Aa^
	7	4.32 ± 0.24 ^Ab^	5.89 ± 1.14 ^Aab^	5.76 ± 0.14 ^Aab^	5.68 ± 1.84 ^Aab^	5.84 ± 1.10 ^Aab^	5.74 ± 1.23 ^Aab^	5.87 ± 1.25 ^Aab^
	14	3.56 ± 1.06 ^Ab^	5.55 ± 0.67 ^Aab^	5.42 ± 1.37 ^Aab^	5.37 ± 1.56 ^Aab^	5.56 ± 1.01 ^Aab^	5.59 ± 0.67 ^Aabc^	5.62 ± 1.11 ^Aabc^
	28	2.18 ± 0.67 ^Bc^	5.21 ± 0.49 ^Aabc^	5.33 ± 1.67 ^Aab^	5.13 ± 0.69 ^Aabc^	5.33 ± 0.86 ^Aabc^	5.37 ± 1.15 ^Aabc^	5.33 ± 0.62 ^Aabc^
	49	ND	4.35 ± 1.36 ^Abcd^	4.86 ± 1.33 ^Aabc^	4.75 ± 0.91 ^Aabcd^	4.91 ± 0.74 ^Aabc^	5.03 ± 1.36 ^Aabc^	4.98 ± 0.50 ^Aabcd^
	63	ND	3.78 ± 0.24 ^Acde^	4.02 ± 0.97 ^Abcd^	4.39 ± 0.88 ^Aabcd^	4.52 ± 1.47 ^Aabcd^	4.53 ± 1.65 ^Aabc^	4.67 ± 0.97 ^Aabcd^
	77	ND	3.01 ± 1.26 ^Ade^	3.22 ± 1.39 ^Acd^	4.08 ± 1.10 ^Abcd^	4.14 ± 0.56 ^Abcd^	4.19 ± 0.74 ^Aabc^	4.29 ± 1.28 ^Aabcd^
	91	ND	2.56 ± 1.57 ^Ae^	2.76 ± 0.69 ^Ad^	3.57 ± 0.84 ^Abcd^	3.88 ± 1.59 ^Abcd^	3.76 ± 1.43 ^Aabc^	3.96 ± 0.94 ^Abcd^
	105	ND	2.39 ± 0.66 ^Ae^	2.23 ± 0.28 ^Ad^	3.09 ± 1.36 ^Acd^	3.36 ± 1.57 ^Acd^	3.14 ± 2.61 ^Abc^	3.47 ± 2.02 ^Abc^
	119	ND	2.17 ± 0.09 ^Ae^	2.26 ± 0.82 ^Ad^	2.75 ± 0.60 ^Ad^	2.84 ± 0.67 ^Ad^	2.88 ± 1.69 ^Ac^	3.09 ± 1.35 ^Ac^
b*	0	14.32 ± 0.63 ^Aa^	14.32 ± 0.63 ^Aa^	14.32 ± 0.63 ^Aa^	14.32 ± 0.63 ^Aa^	14.32 ± 0.63 ^Aa^	14.32 ± 0.63 ^Aa^	14.32 ± 0.63 ^Aa^
	7	16.74 ± 1.23 ^Ab^	16.22 ± 0.32 ^Aab^	16.43 ± 1.84 ^Aab^	15.76 ± 0.88 ^Aab^	15.34 ± 1.94 ^Aab^	15.64 ± 0.74 ^Aab^	15.04 ± 1.74 ^Aa^
	14	20.32 ± 0.89 ^Ac^	17.24 ± 0.87 ^Bab^	16.76 ± 0.33 ^Bbc^	16.87 ± 1.84 ^Bab^	15.98 ± 0.88 ^Bbc^	16.75 ± 2.23 ^Bab^	15.97 ± 1.45 ^Ba^
	28	25.13 ± 1.55 ^Ad^	16.87 ± 1.42 ^Bbc^	17.59 ± 0.91 ^Bc^	17.33 ± 0.51 ^Bbc^	17.02 ± 0.66 ^Bc^	17.23 ± 0.17 ^Bbc^	16.53 ± 1.07 ^Ba^
	49	ND	19.06 ± 0.52 ^Ac^	19.24 ± 1.14 ^Acd^	18.52 ± 1.17 ^Ac^	17.86 ± 1.39 ^Ac^	18.39 ± 1.93 ^Acd^	17.32 ± 0.73 ^Ab^
	63	ND	21.44 ± 1.70 ^Ad^	21.33 ± 0.51 ^Ade^	20.73 ± 0.88 ^Ad^	21.04 ± 2.06 ^Ad^	19.77 ± 0.36 ^Ade^	19.02 ± 2.43 ^Ac^
	77	ND	22.39 ± 1.19 ^Ae^	20.97 ± 0.79 ^ABef^	21.66 ± 1.09 ^Ade^	21.57 ± 0.94 ^Ade^	21.62 ± 0.75 ^Aef^	19.76 ± 0.23 ^Bcd^
	91	ND	23.46 ± 0.53 ^Ae^	22.03 ± 1.52 ^Af^	22.86 ± 1.67 ^Ade^	22.36 ± 1.23 ^Adef^	22.45 ± 0.26 ^Aef^	22.03 ± 1.77 ^Acd^
	105	ND	24.01 ± 0.49 ^Ae^	23.41 ± 0.53 ^Af^	23.47 ± 1.01 ^Aef^	23.77 ± 0.75 ^Aef^	23.64 ± 1.52 ^Afg^	22.87 ± 1.19 ^Ad^
	119	ND	24.64 ± 1.09 ^Af^	24.66 ± 1.19 ^Ag^	24.35 ± 1.15 ^Af^	24.54 ± 0.55 ^Af^	24.23 ± 1.66 ^Ag^	23.76 ± 0.88 ^Ad^

PBMAs, plant-based meat analogs; CK, control group; LCEO, *Litsea cubeba* essential oil; CEO, cinnamon essential oil; LC/CEO, combination of *Litsea cubeba* and cinnamon essential oil nanoemulsions; LCEON, *Litsea cubeba* essential oil nanoemulsion; CEON, cinnamon essential oil nanoemulsion; LC/CEON, combination of *Litsea cubeba* and cinnamon essential oil nanoemulsions; ND, not detected. Data are presented as means ± standard deviation. ^A–C^ Means in the same row followed by different letters are significantly different (*p* < 0.05). ^a–h^ Means in the same column followed by different letters are significantly different (*p* < 0.05).

## Data Availability

The original contributions presented in the study are included in the article, further inquiries can be directed to the corresponding authors.

## References

[1] Rubio N.R., Xiang N., Kaplan D.L. (2020). Plant-based and cell-based approaches to meat production. Nat. Commun..

[2] Bryant C.J. (2022). Plant-based animal product alternatives are healthier and more environmentally sustainable than animal products. Future Foods.

[3] Zhang Q., Liu Y., He C., Zhu R., Li M., Lam H.-M., Wong W.-T. (2023). Nutritional Assessment of Plant-Based Meat Products Available on Hong Kong Market: A Cross-Sectional Survey. Nutrients.

[4] Ahmad M., Qureshi S., Akbar M.H., Siddiqui S.A., Gani A., Mushtaq M., Hassan I., Dhull S.B. (2022). Plant-based meat alternatives: Compositional analysis, current development and challenges. Appl. Food Res..

[5] Andreani G., Sogari G., Marti A., Froldi F., Dagevos H., Martini D. (2023). Plant-based meat alternatives: Technological, nutritional, environmental, market, and social challenges and opportunities. Nutrients.

[6] Singh M., Trivedi N., Enamala M.K., Kuppam C., Parikh P., Nikolova M.P., Chavali M. (2021). Plant-based meat analogue (PBMA) as a sustainable food: A concise review. Eur. Food Res. Technol..

[7] Guyony V., Fayolle F., Jury V. (2023). High moisture extrusion of vegetable proteins for making fibrous meat analogs: A review. Food Rev. Int..

[8] Wang L., Xu J., Zhang M., Zheng H., Li L. (2022). Preservation of soy protein-based meat analogues by using PLA/PBAT antimicrobial packaging film. Food Chem..

[9] Melin P. (2024). Sorbic acid is an efficient preservative in pea-based meat analogues. LWT.

[10] Vila-Clarà G., Vila-Martí A., Vergés-Canet L., Torres-Moreno M. (2024). Exploring the Role and Functionality of Ingredients in Plant-Based Meat Analogue Burgers: A Comprehensive Review. Foods.

[11] Dai Z., Han L., Li Z., Gu M., Xiao Z., Lu F. (2022). Combination of chitosan, tea polyphenols, and nisin on the bacterial inhibition and quality maintenance of plant-based meat. Foods.

[12] Almeida-Souza F., Magalhães I.F., Guedes A.C., Santana V.M., Teles A.M., Mouchrek A.N., Calabrese K.S., Abreu-Silva A.L. (2022). Safety assessment of essential oil as a food ingredient. Essential Oils: Applications and Trends in Food Science and Technology.

[13] Salan L.C., Cropotova J. (2022). An update on effectiveness and practicability of plant essential oils in the food industry. Plants.

[14] Pante G.C., Castro J.C., Lini R.S., Romoli J.C.Z., Almeida R.T.R.D., Garcia F.P., Nakamura C.V., Pilau E.J., Filho B.A.D.A., Machinski M. (2021). Litsea cubeba essential oil: Chemical profile, antioxidant activity, cytotoxicity, effect against Fusarium verticillioides and fumonisins production. J. Environ. Sci. Health Part B.

[15] Qiu Y., Wang Y., Li Y. (2022). Solvent-free microwave extraction of essential oils from *Litsea cubeba* (Lour.) Pers. at different harvesting times and their skin-whitening cosmetic potential. Antioxidants.

[16] Si L., Chen Y., Han X., Zhan Z., Tian S., Cui Q., Wang Y. (2012). Chemical composition of essential oils of *Litsea cubeba* harvested from its distribution areas in China. Molecules.

[17] Wang X.-Y., Li B.-T., Wen Z.-Q. (2023). Volatile constituents of the leaf and fruit essential oils of *Litsea cubeba* (Lour.) Pers. growing wild in Baoshan region, China. Nat. Prod. Res..

[18] Sun C., Cheng X., Yuan C., Xia X., Zhou Y., Zhu X. (2024). *Carboxymethyl cellulose*/Tween 80/*Litsea cubeba* essential oil nanoemulsion inhibits the growth of *Penicillium digitatum* and extends the shelf-life of ‘Shatangju’ mandarin. Food Control.

[19] Sharma S., Barkauskaite S., Jaiswal A.K., Jaiswal S. (2021). Essential oils as additives in active food packaging. Food Chem..

[20] Zhang Y., Liu X., Wang Y., Jiang P., Quek S. (2016). Antibacterial activity and mechanism of cinnamon essential oil against *Escherichia coli* and *Staphylococcus aureus*. Food Control.

[21] Yang K., Liu A., Hu A., Li J., Zen Z., Liu Y., Tang S., Li C. (2021). Preparation and characterization of cinnamon essential oil nanocapsules and comparison of volatile components and antibacterial ability of cinnamon essential oil before and after encapsulation. Food Control.

[22] Zhu Y.-A., Sun P., Duan C., Cao Y., Kong B., Wang H., Chen Q. (2023). Improving stability and bioavailability of curcumin by quaternized chitosan coated nanoemulsion. Food Res. Int..

[23] Li J., Hwang I.-C., Chen X., Park H.J. (2016). Effects of chitosan coating on curcumin loaded nano-emulsion: Study on stability and in vitro digestibility. Food Hydrocoll..

[24] Marhamati M., Ranjbar G., Rezaie M. (2021). Effects of emulsifiers on the physicochemical stability of Oil-in-water Nanoemulsions: A critical review. J. Mol. Liq..

[25] Stoleru E., Brebu M. (2021). Stabilization techniques of essential oils by incorporation into biodegradable polymeric materials for food packaging. Molecules.

[26] Abdalla G., Mussagy C.U., Brasil G.S.A.P., Scontri M., da Silva Sasaki J.C., Su Y., Bebber C., Rocha R.R., de Sousa Abreu A.P., Goncalves R.P. (2023). Eco-sustainable coatings based on chitosan, pectin, and lemon essential oil nanoemulsion and their effect on strawberry preservation. Int. J. Biol. Macromol..

[27] Su R., Guo X., Cheng S., Zhang Z., Yang H., Wang J., Song L., Liu Z., Wang Y., Lü X. (2023). Inactivation of Salmonella using ultrasound in combination with *Litsea cubeba* essential oil nanoemulsion and its bactericidal application on cherry tomatoes. Ultrason. Sonochem..

[28] Kang Z., Chen S., Zhou Y., Ullah S., Liang H. (2022). Rational construction of citrus essential oil nanoemulsion with robust stability and high antimicrobial activity based on combination of emulsifiers. Innov. Food Sci. Emerg. Technol..

[29] Xiong Y., Li S., Warner R.D., Fang Z. (2020). Effect of oregano essential oil and resveratrol nanoemulsion loaded pectin edible coating on the preservation of pork loin in modified atmosphere packaging. Food Control.

[30] Zhou X., Guo X., Chai Y., Li X., Chen L., Feng X. (2024). Superabsorbent whey protein isolates/chitosan-based antibacterial aerogels: Preparation, characterization and application in chicken meat preservation. Int. J. Biol. Macromol..

[31] Liang Z., Veronica V., Huang J., Zhang P., Fang Z. (2022). Combined effects of plant food processing by-products and high oxygen modified atmosphere packaging on the storage stability of beef patties. Food Control.

[32] Wang S., Liu Z., Zhao M., Gao C., Wang J., Li C., Dong X., Liu Z., Zhou D. (2023). Chitosan-wampee seed essential oil composite film combined with cold plasma for refrigerated storage with modified atmosphere packaging: A promising technology for quality preservation of golden pompano fillets. Int. J. Biol. Macromol..

[33] Wei Z., Huang L., Feng X., Cui F., Wu R., Kong Q., Sun K., Gao J., Guo J. (2023). Development of functional, sustainable pullulan-sodium alginate-based films by incorporating essential oil microemulsion for chilled pork preservation. Int. J. Biol. Macromol..

[34] Peng H., Zhang J., Wang S., Qi M., Yue M., Zhang S., Song J., Wang C., Zhang D., Wang X. (2022). High moisture extrusion of pea protein: Effect of l-cysteine on product properties and the process forming a fibrous structure. Food Hydrocoll..

[35] de Oliveira Paulo L.A., Fernandes R.N., Simiqueli A.A., Rocha F., Dias M.M.D.S., Minim V.P.R., Minim L.A., Vidigal M.C.T.R. (2023). Baru oil (*Dipteryx alata* vog.) applied in the formation of O/W nanoemulsions: A study of physical-chemical, rheological and interfacial properties. Food Res. Int..

[36] Bashiri S., Ghanbarzadeh B., Ayaseh A., Dehghannya J., Ehsani A., Ozyurt H. (2020). Essential oil-loaded nanostructured lipid carriers: The effects of liquid lipid type on the physicochemical properties in beverage models. Food Biosci..

[37] Campolo O., Giunti G., Laigle M., Michel T., Palmeri V. (2020). Essential oil-based nano-emulsions: Effect of different surfactants, sonication and plant species on physicochemical characteristics. Ind. Crop. Prod..

[38] Mahdi A.A., Al-Maqtari Q.A., Mohammed J.K., Al-Ansi W., Cui H., Lin L. (2021). Enhancement of antioxidant activity, antifungal activity, and oxidation stability of Citrus reticulata essential oil nanocapsules by clove and cinnamon essential oils. Food Biosci..

[39] Iqbal S.Z., Haider A., Rehman F.U., Cui G., Waseem M., Iqbal M., Khaneghah A.M. (2024). Application of carboxymethylcellulose in combination with essential oils nano-emulsions edible coating for the preservation of kiwifruit. Int. J. Biol. Macromol..

[40] Yin L.-J., Chu B.-S., Kobayashi I., Nakajima M. (2009). Performance of selected emulsifiers and their combinations in the preparation of β-carotene nanodispersions. Food Hydrocoll..

[41] Barradas T.N., de Holanda e Silva K.G. (2021). Nanoemulsions of essential oils to improve solubility, stability and permeability: A review. Environ. Chem. Lett..

[42] Hou P., Pu F., Zou H., Diao M., Zhao C., Xi C., Zhang T. (2019). Whey protein stabilized nanoemulsion: A potential delivery system for ginsenoside Rg3 whey protein stabilized nanoemulsion: Potential Rg3 delivery system. Food Biosci..

[43] Hauptmann A., Handle K.F., Baloh P., Grothe H., Loerting T. (2016). Does the emulsification procedure influence freezing and thawing of aqueous droplets?. J. Chem. Phys..

[44] Liao Z., Wang X., Lu M., Zhong R., Xiao J., Rogers M.A., Cao Y., Lan Y. (2024). Interfacial crystallized oleogel emulsion with improved freeze-thaw stability and tribological properties: Influence of cooling rate. Food Chem..

[45] Mateen A., Singh G. (2023). Evaluating the potential of millets as blend components with soy protein isolate in a high moisture extrusion system for improved texture, structure, and colour properties of meat analogues. Food Res. Int..

[46] Wang W., Zhao D., Xiang Q., Li K., Wang B., Bai Y. (2021). Effect of cinnamon essential oil nanoemulsions on microbiological safety and quality properties of chicken breast fillets during refrigerated storage. LWT.

[47] Zhang Y., Zhou L., Zhang C., Show P.L., Du A., Fu J., Ashokkumar V. (2020). Preparation and characterization of curdlan/polyvinyl alcohol/thyme essential oil blending film and its application to chilled meat preservation. Carbohydr. Polym..

[48] Yuan L., Feng W., Zhang Z., Peng Y., Xiao Y., Chen J. (2021). Effect of potato starch-based antibacterial composite films with thyme oil microemulsion or microcapsule on shelf life of chilled meat. LWT.

[49] Geng L., Liu K., Zhang H. (2023). Lipid oxidation in foods and its implications on proteins. Front. Nutr..

[50] Tóth A.J., Dunay A., Battay M., Illés C.B., Bittsánszky A., Süth M. (2021). Microbial spoilage of plant-based meat analogues. Appl. Sci..

[51] Hamad A., Ramadhan M., Dewi D., Hartanti D. (2020). Preservation potential of lemon basil essential oil on tofu: Development of a natural food preservative. IOP Conf. Ser. Mater. Sci. Eng..

[52] Rusman A.H., Kumar P., Hayat M.N., Abdul M., Adamu G., Ismail-Fitry M.R., Sazili A.Q. (2014). Effect of Sodium Alginate Coating Containing Clove Essential Oil on pH, Cooking Loss, Colour and Sensory Attributes of Refrigerated Rabbit Meat. J. Meat Sci..

[53] Bao Y., Ertbjerg P. (2015). Relationship between oxygen concentration, shear force and protein oxidation in modified atmosphere packaged pork. Meat Sci..

[54] Aguirre-Garcia Y.L., Nery-Flores S.D., Campos-Muzquiz L.G., Flores-Gallegos A.C., Palomo-Ligas L., Ascacio-Valdés J.A., Sepúlveda-Torres L., Rodríguez-Herrera R. (2024). Lactic Acid Fermentation in the Food Industry and Bio-Preservation of Food. Fermentation.

[55] Khan I., Tango C.N., Oh D.H. (2017). Development and evaluation of chitosan and its derivative for the shelf life extension of beef meat under refrigeration storage. Int. J. Food Sci. Technol..

[56] Kaczmarek A.M., Muzolf-Panek M. (2022). Predictive modelling of TBARS changes in the intramuscular lipid fraction of raw ground pork enriched with plant extracts. J. Food Sci. Technol..

[57] Wang L., Yuan M., Sun E., Wu J., Lv A., Zhang X., Guo J., Zhu Y., Guo H., Li X. (2023). Antibacterial food packaging capable of sustained and unidirectional release carvacrol/thymol nanoemulsions for pork preservation. Food Hydrocoll..

[58] Donsì F., Ferrari G. (2016). Essential oil nanoemulsions as antimicrobial agents in food. J. Biotechnol..

[59] Jafarizadeh-Malmiri H., Anarjan N., Berenjian A. (2022). Developing three-component ginger-cinnamon-cardamom composite essential oil nanoemulsion as natural food preservatives. Environ. Res..

[60] da Silva B.D., Rosário D.K.A.D., Weitz D.A., Conte-Junior C.A. (2022). Essential oil nanoemulsions: Properties, development, and application in meat and meat products. Trends Food Sci. Technol..

[61] Xia J., Jiang N., Zhang B., Sun R., Zhu Y., Xu W., Wang C., Liu Q., Ma Y. (2023). Bacterial Changes in Boiled Crayfish between Different Storage Periods and Characterizations of the Specific Spoilage Bacteria. Foods.

[62] Wang Y., Cen C., Chen J., Zhou C., Fu L. (2021). Nano-emulsification improves physical properties and bioactivities of litsea cubeba essential oil. LWT.

[63] (2020). Plant-Based Meat Products.

[64] Falleh H., Jemaa M.B., Neves M.A., Isoda H., Nakajima M., Ksouri R. (2021). Formulation, physicochemical characterization, and anti-*E. coli* activity of food-grade nanoemulsions incorporating clove, cinnamon, and lavender essential oils. Food Chem..

[65] Fevzioglu M., Ozturk O.K., Hamaker B.R., Campanella O.H. (2020). Quantitative approach to study secondary structure of proteins by FT-IR spectroscopy, using a model wheat gluten system. Int. J. Biol. Macromol..

[66] Li L., Ren S., Yang H., Liu J., Zhang J. (2023). Study of the molecular structure of proteins in eggs under different storage conditions. J. Food Process. Preserv..

[67] Wang K., Sun D.-W., Pu H., Wei Q. (2017). Principles and applications of spectroscopic techniques for evaluating food protein conformational changes: A review. Trends Food Sci. Technol..

[68] Zhong Y., Han P., Sun S., An N., Ren X., Lu S., Wang Q., Dong J. (2022). Effects of apple polyphenols and hydroxypropyl-β-cyclodextrin inclusion complexes on the oxidation of myofibrillar proteins and microstructures in lamb during frozen storage. Food Chem..

[69] Huang X., Sun L., Liu L., Wang G., Luo P., Tang D., Huang Q. (2022). Study on the mechanism of mulberry polyphenols inhibiting oxidation of beef myofibrillar protein. Food Chem..

